# Morphometric and Genetic Description of Trophic Adaptations in Cichlid Fishes

**DOI:** 10.3390/biology11081165

**Published:** 2022-08-03

**Authors:** Leah DeLorenzo, Victoria DeBrock, Aldo Carmona Baez, Patrick J. Ciccotto, Erin N. Peterson, Clare Stull, Natalie B. Roberts, Reade B. Roberts, Kara E. Powder

**Affiliations:** 1 Department of Biological Sciences, Clemson University, Clemson, SC 29634, USA; ldelore@g.clemson.edu (L.D.); victoriadebrock@gmail.com (V.D.); 2 Department of Biological Sciences and Genetics and Genomics Academy, North Carolina State University, Raleigh, NC 27695, USA; acarmon@ncsu.edu (A.C.B.); pciccotto@warren-wilson.edu (P.J.C.); erin.peterson@q2labsolutions.com (E.N.P.); cnstull@ncsu.edu (C.S.); nbhodges@ncsu.edu (N.B.R.); rbrober2@ncsu.edu (R.B.R.); 3 Department of Biology, Warren Wilson College, Swannanoa, NC 28778, USA

**Keywords:** craniofacial, quantitative trait loci, geometric morphometrics

## Abstract

**Simple Summary:**

Skull and jaw shape are critical to how an animal eats. The goal of this work was to examine how facial variation evolves and the genetic basis of these changes. We used two species of Lake Malawi cichlids with different facial shapes, one which has evolved to eat prey by suction feeding, a second that bites algae from rocks, as well as hybrid individuals generated by artificial mating of the two species. We found a series of changes in craniofacial structure including the shape of the lower jaw and throat region that impact how animals perform at suction feeding and biting. We then identified genetic regions that regulate these facial shapes. These genetic regions suggested that evolution of the senses, among other traits, may play an important role in facial evolution. Also, evolution of different parts of the face are controlled by distinct genetic regions. Despite this, cichlids that eat similar ways have similar facial shapes, suggesting that the function of jaw movement places certain limits on facial evolution in cichlid fishes. Overall, this work provides insights into how the face evolves, how these changes relate to feeding, and the genes and molecules that regulate craniofacial variation.

**Abstract:**

Since Darwin, biologists have sought to understand the evolution and origins of phenotypic adaptations. The skull is particularly diverse due to intense natural selection on feeding biomechanics. We investigated the genetic and molecular origins of trophic adaptation using Lake Malawi cichlids, which have undergone an exemplary evolutionary radiation. We analyzed morphological differences in the lateral and ventral head shape among an insectivore that eats by suction feeding, an obligate biting herbivore, and their F_2_ hybrids. We identified variation in a series of morphological traits—including mandible width, mandible length, and buccal length—that directly affect feeding kinematics and function. Using quantitative trait loci (QTL) mapping, we found that many genes of small effects influence these craniofacial adaptations. Intervals for some traits were enriched in genes related to potassium transport and sensory systems, the latter suggesting co-evolution of feeding structures and sensory adaptations for foraging. Despite these indications of co-evolution of structures, morphological traits did not show covariation. Furthermore, phenotypes largely mapped to distinct genetic intervals, suggesting that a common genetic basis does not generate coordinated changes in shape. Together, these suggest that craniofacial traits are mostly inherited as separate modules, which confers a high potential for the evolution of morphological diversity. Though these traits are not restricted by genetic pleiotropy, functional demands of feeding and sensory structures likely introduce constraints on variation. In all, we provide insights into the quantitative genetic basis of trophic adaptation, identify mechanisms that influence the direction of morphological evolution, and provide molecular inroads to craniofacial variation.

## 1. Introduction

Understanding the patterns and origins of variation is a key challenge within both developmental and evolutionary biology. A structure with significant morphological diversity is the skull, with variation across and within many clades of vertebrates including fishes [1,2,3], birds [4,5,6], reptiles [7,8], and mammals [9,10,11,12,13]. A critical selective pressure faced by craniofacial structures is trophic niche specialization, with skull morphology directly feeding into biomechanical performance and fitness [14]. These forces shape a complex geometry of the skull, with morphological variation deriving from the cumulative effects of genetics, developmental processes, environmental effects, and functional interactions [15,16,17,18,19,20].

An iconic system for studying morphological variation is cichlid fishes, which have undergone one of the most rapid diversifications in vertebrates [21,22]. The largest radiations of cichlids occurred independently in the African Rift Lakes of Lakes Malawi, Tanganyika, and Victoria, with further radiations occurring throughout smaller lakes and rivers in the African Rift Valley, Central America, and South America [21,23,24,25]. A hallmark of their adaptive radiation is the diversity of craniofacial structures, which are intimately connected to their feeding niche and ecology [2,26,27]. Cichlids, like other teleost fishes, have evolved multiple disparate feeding strategies including suction feeding, biting, and ram feeding, each of which is associated with a suite of phenotypic adaptations [27]. Despite this range of craniofacial morphologies in cichlids, a major ecomorphological axis of variation in cichlids distinguishes two of these strategies, suction feeding and biting [2,28]. On one end of this axis are suction feeders. These animals eat from the water column by generating a high rate of flow into the mouth that overcome any flow in the opposite direction or attempts by mobile prey to swim away [29,30,31,32]. Morphologically, this is accomplished through a large buccal cavity and restricted mouth size that confer an ability to generate pressure differentials in the oral cavity [29,30]. Production of the pressure differential is enhanced through a relatively long mandible that allows quick movements of the jaw [31,33,34,35,36]. Furthermore, large eyes in suction feeders may increase vision to provide an advantage in hunting prey [37], but may also constrain the size of jaw muscles needed for mandible movement [38]. On the alternate end of this morphological spectrum are fishes that feed by scraping/biting attached algae or crushing shelled invertebrates. These fish trade off speed in mandible movements for power with jaw closing, primarily conferred by a shorter lower jaw [31,33,34,35].

Cichlids from independent radiations have undergone similar divergences in craniofacial morphology between fish that suction feed versus bite [28,39,40], and this trend extends more broadly across fishes as well [41,42,43]. This pattern suggests that genetic, developmental, or functional constraints are limiting or biasing the direction of morphological evolution in the skull [44,45,46,47]. Furthermore, specific regions of the face may evolve autonomously as independent modules, or as coordinated units [47]. For example, coordinated changes could be driven by “supergene” regions [48,49,50] or biomechanical demands of ecological niches may cause convergent evolution of form (e.g., [51]). Previous analysis of the strengths and patterns of covariation (i.e., modularity) in cichlid craniofacial structures [52] found that the pre-orbital and post-orbital regions act as independent modules. However, studies in mammals, birds, and the Archosaur group have identified modularity within the skull based on developmental origins [53,54,55] or function and articulation [54,56]. A full understanding of the patterns of morphological variation, as well as the number and effects of genes that underlie these shapes, is necessary to clarify which aspects of head anatomy demonstrate covariation, have increased evolutionary flexibility, or are simpler versus more complex phenotypes.

Here, we use two species of cichlids to investigate the adaptation of craniofacial morphology and the genetic basis of this variation. Both *Labidochromis caeruleus* and *Labeotropheus trewavasae* live in rocky habitats of Lake Malawi, but feed by suction feeding and scraping, respectively [26]. Fishes of the *Labidochromis* genus are typically insectivores that suction feed or pluck their prey from the water column [26]. Alternatively, fishes of the *Labeotropheus* genus strictly feed by biting algae that is attached to rocky substrates [26]. We had three goals of this study. First, we sought to quantify a series of morphological adaptations in the lateral and ventral craniofacial head anatomy in these species, relating these morphologies to feeding mechanics of these fishes. Second, we aimed to identify the genetic basis underlying these morphologies utilizing quantitative trait loci (QTL) mapping in a population of *Labidochromis* × *Labeotropheus* F_2_ hybrids. The resulting data allowed us to use gene ontology (GO) term analysis of candidate genes to uncover enriched molecular mechanisms and pathways that may influence craniofacial morphological diversity. Third and finally, we sought to determine if these traits are inherited independently or as sets of traits through examination of both morphological and genetic data, with important implications for the direction and patterns of morphological evolution of these craniofacial structures. Overall, these data should elucidate genetic factors that influence diversity in trophic adaptations of the craniofacial skeleton and drive major morphological variation in the skull.

## 2. Materials and Methods

### 2.1. Fishes and Pedigree

All work was completed under animal protocol 140-101-O approved by the Institutional Animal Care and Use Committee (IACUC) at North Carolina State University. All animal rearing and breeding described below occurred in laboratory aquatic systems; original stocks were wild caught in Lake Malawi and subsequently bred in the aquarium trade. Lab aquarium conditions were designed to mimic the natural environment of these fishes in Lake Malawi in terms of water quality parameters and substrate in the aquaria to mimic a rocky habitat. Three distinct groups were analyzed for this study. The first group was a set of 10 *Labidochromis caeruleus* specimens, hereafter referred to *Labidochromis* parentals as they represent the shape of the granddam used in the cross described below. The second group was a set of *Labeotropheus trewavasae* specimens, hereafter referred to as *Labeotropheus* parentals as they represent the shape of the grandsire used in the cross described below. The third set of animals were F_2_ hybrids generated from a cross between *Labidochromis* and *Labeotropheus*, generated as described below. A single *Labidochromis* female was crossed with a single *Labeotropheus* male to create one F_1_ family. These F1 siblings mated with each other to produce a hybrid F_2_ population of 447 fishes. Fish were reared in lab aquaria under standard feeding with flake food for five months, at which time they were euthanized with buffered MS-222 for morphological analysis. F_2_ hybrids ranged in weight from 0.35–3.19 g (mean = 1.21 g, standard deviation = 0.47 g). Sex in Lake Malawi cichlids is genetically determined by multiple loci, with the specific interaction dependent on the species [57,58]. In the F_2_ hybrids, sex was called based on gonad dissection for 354 of the 447 animals, with 92.9% being male. Given this imbalance, specimens were not analyzed separately for males versus females. Lateral and ventral images of each specimen were taken using an Olympus digital camera under standardized lighting conditions in a lightbox. A color standard and scale were included in each picture.

### 2.2. Linear Measures of Head Shape Variation

**Figure 1:**
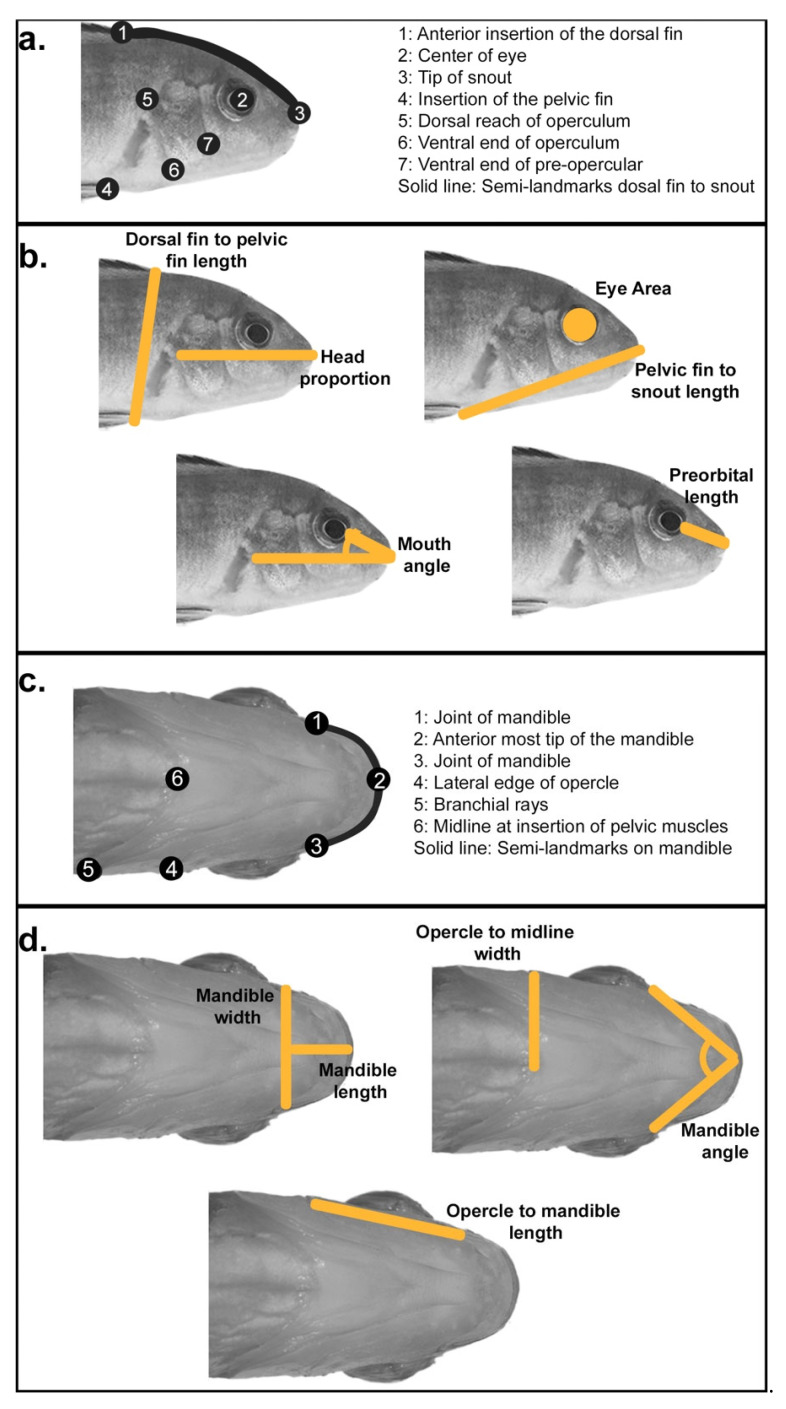
Measures used to analyze lateral and ventral head shape. (**a**,**c**) Geometric and (**b**,**d**) linear measures were used to assess head shape changes with functional implications for feeding biomechanics.

Measures were taken from photographs of 10 parental specimens per species of *Labidochromis* and *Labeotropheus,* and either 447 F_2_ hybrids for lateral analysis or 319 F_2_ hybrids for ventral analysis. From photographs of the lateral body, we measured standard length (snout to caudal peduncle), head length (snout to opercle), head depth (anterior insertion of the dorsal fin to the insertion of the pelvic fin), length from the snout to the insertion of the pelvic fin, preorbital length (snout to anterior edge of the eye), eye diameter, and mouth angle (Figure 1b). Eye area was calculated from eye diameter measurements using the formula π*(diameter/2)^2^. Measures of the ventral anatomy included mandible width, mandible length, width from the posterior of the opercle to midline, length from the posterior of the opercle to the joint of the mandible and palatoquadrate, and mandible angle (Figure 1d). Measurements were taken using ImageJ software (version 2.0.0) [59,60] as number of pixels and were then converted into centimeters using the scale in each photo. To remove the effects of allometry, all measures were normalized to the standard length of each specimen. After linear regression to standard length measurements, the resulting residual values were used for further analysis. This size correction was carried out on a dataset with both parental species and their hybrids. Statistical analyses were conducted in R (version 3.5.2) [61], including ANOVAs followed by Tukey’s honest significant difference post-hoc tests (Table S1), and Pearson’s correlations (Table S2).

### 2.3. Geometric Morphometric Shape Analysis

Geometric morphometric shape analysis was used to further quantify head shape variation. A series of homologous landmarks were chosen highlighting lateral and ventral craniofacial anatomy important to feeding mechanics (Figure 1a,c). In both cases, we only analyzed one side of the specimen, avoiding the side in which there were body dissections posterior to the pectoral fins. Landmark positions were recorded from photos using the tpsDig2 software package (version 2.31) [62]. These data were uploaded into the R package *geomorph* (version 3.1.3) [63], in which Procrustes superimposition was used to remove variation due to size, rotation, and position of landmarks to leave variation only due to shape. As with the linear data, the effects of allometry were removed through size correction and regression of shape on standard length. One step of geometric morphometric analysis is a principal component analysis that reduces dimensionality of the morphological data into principal component (PC) scores. The geometric morphometric analyses described above were conducted on a dataset including both parental species and their hybrids. Statistical significance in overall shape among parentals and hybrids was confirmed by calculating Procrustes distances between mean shapes of the three groups using the *procD.lm* function in *geomorph*, followed by pairwise comparisons using the *pairwise* function in *geomorph*. Statistical analyses for individual principal component scores were conducted in R (version 3.5.2) [61], including ANOVAs followed by Tukey’s honest significant difference post-hoc tests (Table S1). Statistical strength and patterns of modularity among landmarks was determined using the *modularity.test* function in *geomorph* using 9999 iterations, omitting semi-landmarks as these potentially were in more than one module, and a priori landmark groupings based on facial regions, development, and function (further detailed in Figure S4).

### 2.4. Genotyping with ddRAD Sequencing

Genomic DNA was extracted from caudal fin tissue using DNeasy blood and tissue kits (Qiagen). RADseq libraries were prepared as previously described [64], including double digestion and indexing, then sequenced on Illumina Hiseq with 100 bp paired end reads (North Carolina State University Genomic Sciences Laboratory core facility). The program process_radtags (Stacks, version 2), was used to process raw sequencing data including demultiplexing, truncating reads to 150 bp, and filtering of low-quality reads. Processed reads were aligned to the *Maylandia zebra* UMD2a reference genome using BWA with the mem algorithm. The programs pstacks, cstacks, and sstacks (Stacks, version 1) were used to identify and catalogue RAD markers in the parental and F_2_ hybrid samples. Finally, markers with alternative alleles in the parental species were called as AA or BB genotypes using the program genotypes (Stacks, version 1), requiring a minimum stack depth of 3 to export a maker in a specific individual. The A allele was inherited from the *Labidochromis* granddam and the B allele from the *Labeotropheus* grandsire.

### 2.5. Generation of the Linkage Map

The genetic map was generated using the package R/qtl [65] and in-house R scripts available at https://github.com/kpowder/Biology2022 (accessed on 15 June 2022). Markers were first sorted into linkage groups according to their position in the reference genome for another Lake Malawi cichlid, *Maylandia zebra*, *M. zebra* UMD2a assembly. Markers were removed from the dataset if they were located on unplaced scaffolds with more than 40% of missing data, or in linkage groups with more than 20% missing data. A chi-square test was performed on the remaining markers using the geno.table function. Those markers with a distorted segregation pattern and a Bonferroni-corrected *p*-value < 0.01 were discarded from the dataset. The initial map was generated based on estimated pairwise recombination frequencies using est.map and est.rf functions. Markers in linkage groups that were not initially flagged as misplaced were removed if they increased the size of the map by at least six centimorgans (cM) and flanking markers were < 3 Mb apart. Markers that were in unplaced scaffolds were integrated into a linkage group if they had a recombination frequency < 0.15 with at least five markers from that linkage group. Any other markers that were in unplaced scaffolds that did not meet the above criteria were removed. If markers had irregular relationships between their recombination frequency and position in the genetic map, they were rearranged manually to minimize crossover events; these are likely due to being located in structural variants or misassembled sections of the reference genome. Genotyping errors were identified using the function calc.errorlod and set as missing data if they had a LOD score of ≥ 3. The linkage map was refined with a non-overlapping window algorithm that selected one marker in a 2 cM window with the least amount of missing data. Finally, the function est.map was used to estimate the final map and the maximum likelihood estimate of the genotyping error rate (0.0001). The final map was 1239.5 cM in total size, with 22 linkage groups, 1180 total markers, and 42–81 markers per each linkage group.

### 2.6. Quantitative Trait Loci (QTL) Mapping

We conducted multiple-QTL mapping (MQM) using the R/qtl package [65,66,67] following [68]. Scripts are described and available in [69]. First, an initial scan for QTL was carried out using the onescan function in R/qtl [65]. Putative QTL with a LOD approaching or above 2.5 were used to build a more robust statistical model. The MQM method uses these putative QTL as cofactors in follow-up scans and verifies each cofactor by backward elimination. The use of cofactors in the final model aids in the accurate detection of QTL and assessment of their effects [68]. The statistical significance of each QTL was determined using 1000 permutations on the final model. For QTL peaks meeting 5% (significance) or 10% (suggestive) level, 95% confidence intervals were calculated using Bayes analysis. Details of QTL mapping including cofactors used in the model, significance levels, confidence intervals, and allelic effects are in Table S3. QTL analysis was conducted on both linear and geometric morphometric measures of shape for both lateral and ventral anatomy.

### 2.7. Candidate Gene Annotation and Enrichment Analysis

Markers are named based on contig and nucleotide positions in the *Maylandia zebra* reference genome, *M. zebra* UMD2a assembly. Gene symbols, ID, and chromosomal positions for candidate genes in each QTL interval were retrieved from the NCBI genome data viewer (https://www.ncbi.nlm.nih.gov/genome/gdv (accessed on 15 June 2022)) gene track for *M. zebra* annotation release 104. If the upper and lower limits of a QTL interval were mapped to unplaced scaffolds, the closest marker that mapped to a placed scaffold was used to determine candidate gene information. Gene names for each candidate were retrieved using the NCBI gene ID and the Database for Visualization and Integrated Discovery (DAVID) [70].

Gene ontology (GO) term enrichment analysis was performed with the functional annotation tool in the Database for Visualization and Integrated Discovery (DAVID) [70,71]. NCBI gene ID (entrez gene ID) for candidate genes in QTL intervals were used as a query. Analysis was run for each individual trait, pooling multiple QTL as applicable, as well as bulk analysis of all lateral QTL and all ventral QTL. A *p*-value of 0.05 with a Fishers exact probability test was used to denote significance for terms in GO analysis.

## 3. Results and Discussion

### 3.1. Lateral Head Shape Variation

**Figure 2:**
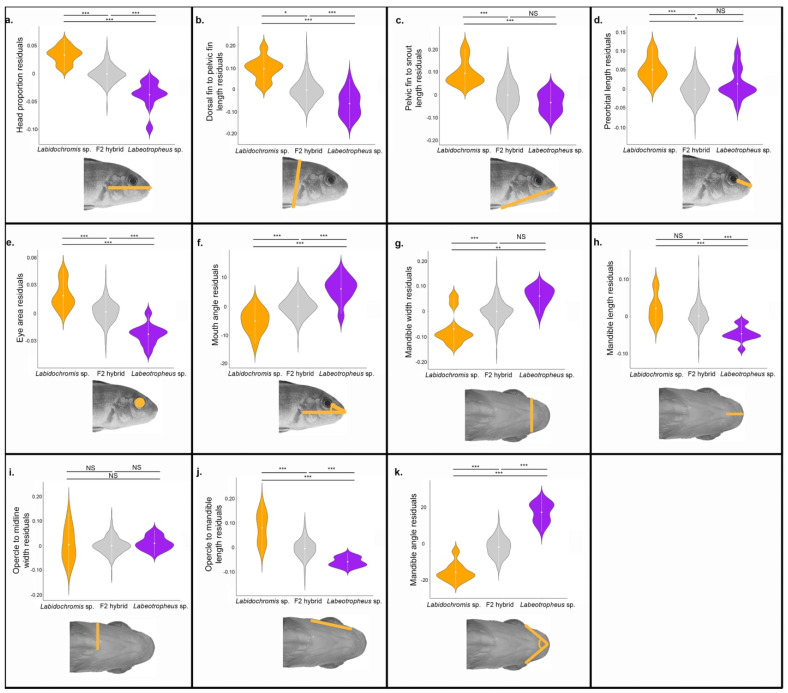
Phenotypic differences among *Labidochromis* sp., *Labeotropheus* sp., and their F_2_ hybrids. Phenotypes measured are indicated by the illustration and include (**a**) head proportion, measured as head length/standard length, (**b**) dorsal-to-pelvic fin length, (**c**) snout-to-pelvic fin length, (**d**) length of the preorbital region of the head, (**e**) eye area, (**f**) mouth angle, (**g**) mandible width, (**h**) mandible length, (**i**) opercle-to-midline width, (**j**) length from the opercle to the mandible, and (**k**) angle formed from posterior ends of the mandible to the midline. Significance in violin plots is based on ANOVA analysis followed by Tukey’s HSD (data in Table S1; *p*-values indicated by * <0.05, ** <0.01, *** <0.005, NS > 0.05).

Lateral skull shape is distinct between parental species *Labidochromis* and *Labeotropheus* for all linear measures (Figure 2a–f and statistics provided in Table S1). Their F_2_ hybrids are largely intermediate in phenotype, though in some cases such as length of the preorbital region (Figure 2d) surpass the range of the parental species. *Labidochromis* fish have an overall longer and deeper head than *Labeotropheus* given a similar body size. Specifically, *Labidochromis* compared to *Labeotropheus* parentals have an increased proportion of the body that is the head (*p* < 1e−7, Figure 2a), a longer distance between the dorsal fin and pelvic fin (*p* < 1e−7, Figure 2b), and larger eye (*p* < 1e−7, Figure 2e). Furthermore, the mouth of *Labidochromis* fish is angled towards the front, rather than towards the ventral side of the body as in *Labeotropheus* (*p* < 1e−7, Figure 2f). Finally, *Labidochromis* showed an increased length between the snout and pelvic fin (*p* = 2.2e−6, Figure 2c). Coupled with a more modest, though still significant, enlargement of the preorbital region (*p* = 0.018, Figure 2d), this suggests that the opercular region of *Labidochromis* fishes is also distinctly larger than in *Labeotropheus*.

**Figure 3:**
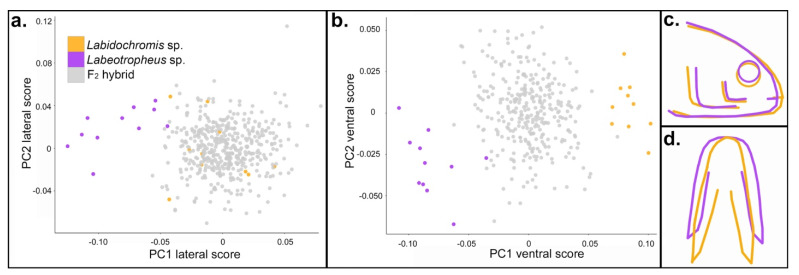
Geometric morphometric phenotypes among parentals and hybrids. Multivariate analysis of shape quantifies differences in overall morphology in the (**a**,**c**) lateral and (**b**,**d**) ventral anatomy. Shapes described by each principal component are detailed in the text and visualized in Figure S1. Average shape (**c**,**d**) of *Labidochromis* sp. (orange) and *Labeotropheus* sp. (purple) based on (**a**,**b**) highlights phenotypic variation between alternate feeding strategies.

Geometric morphometrics provided more detailed insights into shape differences, including within the opercular region of the head. Global lateral shape is statistically distinct among *Labidochromis* sp., *Labeotropheus* sp., and their F_2_ hybrids when calculated as Procrustes distances between mean shapes for each group (*p* = 1e−4 for all pairwise comparisons between *Labidochromis* parentals, *Labeotropheus* parentals, and F_2_ hybrids, Figure 3a). Overall, *Labeotropheus* has a shorter pre-opercle region, opercular region, and dorsally shifted eye than in *Labidochromis*, leading to a compressed craniofacial region with a steeper profile (Figure 3c).

The first five principal components (PCs) described (75.2% total shape variation [TSV]) in lateral shape (Figure 3a,c and Figure S1). PC1 lateral (22.4% TSV) differentiated the two parental species (*p* < 1e−7). The *Labidochromis* species associated with a positive PC1 lateral score that describes a longer head with a more posterior eye placement (Figure 3a,c and Figure S1b). Based on linear measures, this shift in eye position is due to both a larger preorbital region (Figure 2d) and a larger eye area (Figure 2e). As suggested by linear measures, PC1 lateral shape differences show that *Labidochromis* has a larger opercular region, while the operculum in *Labeotropheus* only extends about halfway between the eye and insertion of the pelvic fin (Figure 3c). PC2 lateral, PC3 lateral, and PC4 lateral shape were not significantly different between the parentals (*p* = 0.071, *p* = 0.99, and *p* = 0.77, respectively, Table S1) and thus represent shape variation largely present in the F_2_ hybrids. PC2 lateral (17.2% TSV) predominantly described the relative length of the head, with a negative PC2 lateral score characterizing head anatomy that has a longer profile from snout to dorsal fin and a pelvic fin that is inserted closer to the opercle (Figure S1c). PC3 lateral (13.3% TSV) depicted coordinated changes in both head length and depth, with a negative score representing a deep, short head with a steep craniofacial profile and reduced opercular region (Figure S1d). Notably, a steep craniofacial profile in cichlids (see solid line in Figure 1a) has been associated with an ability for the skull to withstand increased biting forces [72]. PC4 lateral (11.4% TSV) describes differences in the dorsal–ventral depth of the opercular region, as well as the dorsal–ventral positioning of the eye (Figure S1e). Finally, PC5 lateral (11.0% TSV) distinguishes the two parental species (*p* = 0.012). *Labidochromis* parentals are associated with a more negative PC5 lateral score and a reduced opercle bone (Figure S1f).

### 3.2. Ventral Head Shape Variation

Compared to *Labeotropheus*, *Labidochromis* parental fish have a decreased mandible width (*p* < 1e−7, Figure 2g), increased mandible length (*p* = 4e−7, Figure 2h), and longer length of the opercular region (*p* < 1e−7, Figure 2j). Mandible angle assesses the relative proportions of the lower jaw, with an increased measure indicating increased width, decreased length, or both, in the case of *Labeotropheus* (*p* < 1e−7 compared to *Labidochromis*, Figure 2k). These shape changes combine with a similar width at the opercle (*p* = 0.93), Figure 2i), the only measure that was not distinct between parentals. This results in a more triangular ventral shape for *Labidochromis* and a more rectangular shape for *Labeotropheus* parentals (Figure 3d).

Relative mandible length and width also dominated geometric morphometric analysis of the ventral skeleton. Overall ventral shape is statistically distinct among *Labidochromis* sp., *Labeotropheus* sp., and their F_2_ hybrids when calculated as Procrustes distances between mean shapes for each group (*p* = 1e−4 for all pairwise comparisons between *Labidochromis* parentals, *Labeotropheus* parentals, and F_2_ hybrids, Figure 3b). The *Labidochromis* ventral shape features a narrow mandibular shape, relatively wide opercle region, and overall longer face compared to *Labeotropheus* (Figure 3d).

The first three ventral principal components cumulatively describe 76.2% TSV in ventral craniofacial anatomy. PC1 ventral describes 43.6% TSV, with the parental species defining the extremes (*p* < 1e−7, Figure 3b), emphasizing the relevance of this single metric of shape variation on global shape variation between the species. *Labidochromis* parents are associated with a positive PC1 ventral score and a narrower, arched mandible versus the wide and flat mandible shape of *Labeotropheus* (Figure 3b,d and Figure S1g). PC2 ventral (18.7% TSV) is also distinct between parentals (*p* = 8.2e−5, Figure 3b), reflecting the biological relevance of this axis of shape variation. *Labidochromis* and positive scores are associated with a narrow mandible, increased distance of the opercular region, and pectoral fin musculature shifted to the anterior (Figure S1h). PC3 ventral (13.9% TSV) describes relative mandible length without an accompanying change in the width (Figure S1i) and is not significantly different between *Labidochromis* and *Labeotropheus* parentals (*p* = 0.31).

Combining both lateral and ventral shape variation demonstrates the multiple ways *Labidochromis* and *Labeotropheus* have craniofacial biomechanics that are adapted to their feeding niches. *Labidochromis* sp. pluck or suction feed insects within Lake Malawi [26]. Their longer mandibles (Figure 2h) allow more velocity transmission during jaw movement [35], critical for capturing mobile prey. This is combined with a narrow mandible (Figure 2g) that opens into a longer and wider opercular and buccal region (Figure 2i,j), forming a triangular ventral shape (Figure 3d). The large expansion possible in the buccal cavity of *Labidochromis* causes high velocity and acceleration of water flowing into the mouth, containing the invertebrate prey; this water flow is increased by a narrow mouth opening (Figure 2g and Figure 3d) [29,73,74]. On the other hand, *Labeotropheus* sp. are herbivorous grazers that scrape or shear immobile algae from rocks or other substrate using their mandible [26]. The short mandible (Figure 2h) of *Labeotropheus* represents a tradeoff of speed of jaw movement for high transmission of force with jaw closing [35]. This is combined with a downturned mouth (Figure 2f) and a short, wide, and flat preorbital and mandibular region (Figure 2d,g and Figure 3c,d). Together, these are thought to enhance foraging efficiency for *Labeotropheus* by providing a large oral area and structures that are used as a fulcrum to leverage attached algae from their substrate [26].

### 3.3. Genetic Basis of Head Shape

**Figure 4:**
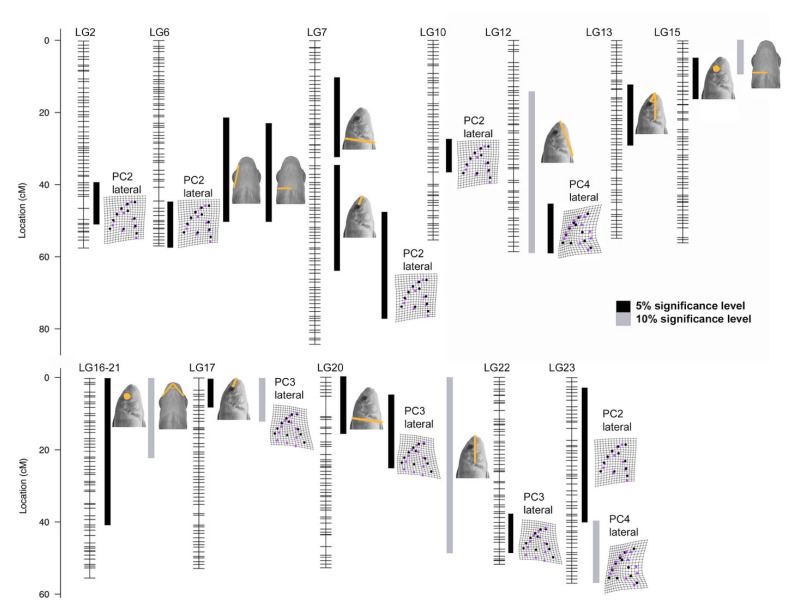
Quantitative trait loci (QTL) mapping identifies 23 intervals associated with head shape variation in hybrids of *Labidochromis* and *Labeotropheus*. Each linkage group (LG, i.e., chromosome) is indicated with genetic markers noted by hash marks. The phenotype related to each QTL region is indicated by illustrations. Black bars are significant at the 5% genome-wide level, while gray bars are suggestive, meeting the 10% genome-wide level. Bar widths indicate 95% confidence interval for the QTL, as calculated by Bayes analysis. QTL scans at the genome and linkage group level are in Figures S2 and S3. Details of the QTL scan including markers and physical locations defining each region are in Table S3.

Quantitative trait loci (QTL) mapping was used to assess the genetic architecture that underlie these adaptive morphologies. We mapped 11 lateral and 8 ventral traits including both linear measures of shape (Figure 2) and principal component scores from geometric morphometric analysis (Figure 3 and Figure S1). This identified 23 genetic intervals that contribute to phenotypic differences in head shape in *Labidochromis* × *Labeotropheus* hybrids (Figure 4, Figures S2 and S3, and Table S3). Between one and three QTL mapped to 12 of the 22 linkage groups. These QTL explained 3.3–7.0% of the total variation for each trait (Figure S3 and Table S3), indicating that each of these traits is controlled by many genes of small effects. Even for the trait with the most QTL, PC2 lateral shape, the five QTL combine to explain only 23.8% of the total coordinated variation (Figure S3) in head length, craniofacial profile, and pelvic fin insertion (Figure S1c). The allelic effects within this QTL (Figure S3) further suggest a complex genetic architecture, with the allele inherited from the *Labidochromis* parent contributing to a higher PC2 lateral score for QTL on LG7 and LG10, the *Labeotropheus* allele associated with a higher value for the QTL on LG2, and heterozygous animals having the largest PC2 lateral score for the QTL on LG6 and LG23. Given that cichlid species continue to segregate and exchange a set of ancestral polymorphisms [75,76,77,78,79], this genetic variation is all likely to contribute to craniofacial divergence and feeding adaptation within the cichlid flock.

While QTL were distributed across linkage groups, seven linkage groups had overlapping QTL intervals (Figure 4 and Table S3). Four of these overlapping regions included a linear measure and a principal component from geometric morphometrics, where the principal component also includes variation in that linear measure. For instance, there are three overlapping QTL intervals on LG20 which describe relative head length, depth of the head from the dorsal fin to the pelvic fin, and PC3 lateral shape (Figure 4). PC3 lateral shape includes major variation in the anterior–posterior length and dorsal–ventral depth of the head (Figure S1d), explaining why these phenotypes map to a common genetic interval. Likewise, preorbital length varies in both PC2 lateral and PC3 lateral shape (Figure S1c,d). QTL for the preorbital region overlap with QTL for PC2 lateral and PC3 lateral on LG7 and LG17, respectively (Figure 4 and Table S3). Finally, the length of the pelvic fin insertion point to the tip of the snout is part of PC4 lateral shape (Figure S1e), and QTL for these traits overlap on LG12 (Figure 4 and Table S3).

### 3.4. Patterns of Covariation and Modularity

To understand the genetic, developmental, or functional factors that may be limiting morphological variation, we examined the patterns of covariation within our data using three different approaches.

We first examined the degree of covariation among our linear measures of shape. Aside from effects of allometry (i.e., correlation with standard length, Table S2), no phenotypes showed correlation (0.8 < Pearson’s r coefficient < −0.8) with each other in the F2 hybrids. Covariation for each pair of phenotypes ranged from −0.65 to 0.78 with a mean of 0.027 (Table S2). This suggests that the morphological traits are largely inherited as modular units rather than as a set of coordinated phenotypes.

Second, we formally tested patterns of modularity using our geometric morphometric landmarks (Figure S4) and hybrid dataset. For the lateral skeleton, none of our modularity models were significant, with *p* = 0.273–0.977 for models based on pre/post orbital regions, developmental origins, and function (respiratory versus vision versus feeding). The ventral anatomy had a *p*-value of 0.0508 for a model assessing pre- versus post-orbital regions, though we note the model cannot differentiate between modules based on these facial regions, developmental origins (anterior versus posterior neural crest streams), and respiratory versus feeding function. While our data could not confirm previous analysis of the cichlid lateral craniofacial anatomy that identified pre-orbital and post-orbital regions acting as independent modules [52] in lateral facial structures, this may be due to the sample size, or the different locations and number of landmarks used.

Finally, we examined the degree to which morphological traits had a common versus distinct genetic basis. A common genetic basis, observed here as overlapping QTL, may also lead to coordinated changes in shape. We noted linkage groups that have overlapping QTL for both lateral and ventral shape variation. LG6 contains a QTL cluster for PC2 lateral shape, opercle-to-mandible length, and opercle-to-midline ventral width (Figure 4 and Table S3). Genetic intervals associated with eye area overlap with opercle-to-midline width on LG15 and mandible angle on LG16-21 (Figure 4 and Table S3); for all these QTL, the allele inherited from *Labidochromis* increases each of these measurements (Figure S3, Table S3). This common genetic basis, and even sometimes common allelic effects, indicate that a single gene or linked genes in this interval may have pleiotropic effects on feeding adaptations. However, the fact that phenotypes were largely controlled by distinct QTL and showed minimal correlations (Table S2) means that distinct feeding morphologies could theoretically evolve independently and recombine into new patterns. This modular pattern would increase the morphological variability possible in cichlids (i.e., be more evolvable) [47,80,81,82,83]. Despite this, three independent, large-scale radiations of cichlids in the African Rift Lakes have generated animals with comparable trophic specializations that share remarkable similarities in their craniofacial morphologies [28,39,40]. Thus, despite largely being independent in terms of genetic structure, morphological disparity is constrained. Our data suggest this is predominantly due to functional demands of feeding and strong natural selection on feeding performance, rather than a genetic constraint [84,85,86].

### 3.5. Gene Ontology (GO) Analysis

To begin to understand the trends and molecular pathways enriched in these candidate genes, GO analysis was performed on candidate genes within QTL intervals (Table S4). Members of the Wnt signaling pathway were significantly enriched (*p* = 0.046, Table S5) for mouth angle including the secreted Wnt antagonist *sfrp5* [87,88], the beta-catenin interacting gene *lzts2a* [89], and the deubiquinitase *zranb1*, which alters Wnt signaling activity [90,91]. We note this is only a single QTL on LG13. There is a strong relationship between the mouth angle and the steepness of the craniofacial profile (see solid line in Figure 1a), with a shallow profile leading to a narrow mouth angle and jaw facing forward. Alternatively, a steep profile is associated with *Labeotropheus* sp. [92], an increased mouth angle (Figure 2f), and ventrally angled jaws. Wnt signaling plays a pivotal role in shape of the craniofacial profile, with increased Wnt signaling causing a retention of larval phenotypes and a steep facial profile in cichlids [92,93]. Based on the function of Wnt signaling in craniofacial development across vertebrates, this is likely through alteration of cellular proliferation and outgrowth [4,92,94,95,96] and precocious bone deposition [92,97,98].

Four traits are statistically significant for changes in potassium transport: head proportion (*p* = 0.018, including various potassium voltage-gated channels such as *kcnc4*, *kcnd3*, *kcng1*, and *kcnk15*), the distance between the dorsal and pelvic fins (*p* = 0.018, including *kcna1a*, *kcnc1b*, *kcnc4*, and *kcnd3*), PC2 lateral shape (*p* = 0.031, including solute transporters *slc9a6a*, *slc12a2*, *slc13a2*, and *slc34a2b*), and PC4 lateral shape (*p* = 0.024, including multiple loci related to sodium/potassium-transporting ATPase subunit alpha-1 and *atp1b1b*) (Table S5, which has a full list of enriched genes). This common signal for head proportion and dorsal-to-pelvic fin length is likely driven by the fact that these traits have an overlapping QTL on LG20, though the genes included are not entirely overlapping. Furthermore, both PC2 lateral (Figure S1c) and PC4 lateral (Figure S1e) include variation in both of these linear measures. Potassium could have numerous influences on craniofacial morphology as this mineral regulates cell proliferation [99], chondrogenesis [100], osteoclast [101] and osteoblast [100,102] differentiation, and bone mineralization [100,102]. Potassium can also influence pathways critical for facial and bone development such as Bmp signaling [94,100,103], which is also associated with mandibular adaptation in cichlids [35]. Finally, mutation of potassium channels can lead to a series of developmental syndromes that include craniofacial morphologies that mimic evolved variation in cichlids. For example, Andersen–Tawil syndrome is characterized by a broad facial width and mandibular hypoplasia [104,105,106], while Birk–Barel syndrome results in a narrow forehead, micrognathia, and cleft or high-arched palate [105,107] (see Figure 2g–i for comparable phenotypes in cichlids).

It is perhaps unsurprising that eye area was enriched for the GO terms olfaction and sensory transduction (*p* = 1.25e−6 and *p* = 5.5e−5, respectively, Table S5), given the common developmental origin of sensory structures [108,109]. However, both mandible angle and a combined analysis of all ventral skeletal morphologies were also enriched for genes associated with these terms (*p* = 3.1e−4 to *p* = 2.26e−8, Table S5). These include various olfactory receptors, as well as genes such as cone rhodopsin subunits and *myo3b*, which mediates mechanosensory neuron response [110]. This may be due to coordinated adaptations for feeding strategies as olfaction and sight are important for identifying mobile prey prior to suction feeding [37,111,112]. However, this may also be due to functional and spatial constraints, wherein a narrow face or large jaw musculature restricts the space available to develop large eyes [38].

### 3.6. Candidate Genes in QTL Intervals

More work is needed to narrow down and determine the specific effects of candidate genes within QTL intervals (Table S4), for instance through fine mapping of intervals that contain hundreds of genes. That said, we highlight in Table 1 several candidate genes for QTL intervals associated with craniofacial development, such as members of Fgf, Wnt, Bmp, and Hedgehog signaling pathways. We discuss below some of these genes, including potential ways in which these genes may mediate the observed phenotypic variation between *Labidochromis* and *Labeotropheus.* We focus this discussion on two genes (*ptch1* and *crocc2*) that have been previously associated with facial variation in cichlids, and two developmental processes (bone remodeling and neural crest cell migration) that highlight the varied ways craniofacial shape can be altered.

Two of our QTL intervals contain genes previously associated with trophic adaptation in cichlid fishes. First is the gene *ptch1* on LG12, within the QTL for pelvic fin to snout length. Regulatory variation in *ptch1* in cichlids has previously been associated with the relative proportions of the mandible, dermal bone development, and the trade-off between speed and power of jaw movements [113]. The mandible variation regulated by *ptch1* includes the length of this bone, which would directly affect the snout length measured here. *Ptch1* may also affect facial length through outgrowth at the midline of the face. Hedgehog signaling, including *ptch1*, is associated with cell survival and coordinated changes in the bones of the upper face and the brain [114,115,116].

Primary cilia are essential for Hedgehog signaling, and the second example is related to a different aspect of cilia function. Primary cilia have been proposed as a critical signaling center for detection of shear stress and mechanotransduction [117]. As cichlids feed by suction feeding or biting (i.e., like *Labidochromis* and *Labeotropheus*, respectively), this stimulates bone remodeling and variation in the overall length and profile of the lateral craniofacial shape [92], similar to PC2 lateral variation in this study. The LG23 QTL for PC2 lateral shape includes the gene *crocc2*, a structural component of primary cilia. In cichlids, coding variation in *crocc2* is associated with the ability to have this mechanic response to feeding, as well as with rates of bone deposition [118]. Thus, PC2 lateral shape may at least partially occur through bone remodeling based on feeding biomechanics.

Shape changes in cichlid facial structures may reflect alteration of late developmental events, such as bone remodeling, or early events in craniofacial development. Within the QTL on LG7 for the distance between the dorsal fin and pelvic fin are three components of semaphorin signaling. Members of this signaling pathway—including candidate genes *sema3a*, *sema3d*, and *sema3e*—are critical regulators of migration of neural crest cells, the precursors of much of the facial skeleton [119,120]. In particular, semaphorin activity controls the cell cycle in migratory neural crest cells [121] and their route of migration [122,123,124]. Given the path of neural crest cell migration from the dorsal side of the neural tube to the ventral pharyngeal arches, alteration of semaphorin signaling may result in more or fewer cells in ventral regions and thus affect overall dorsal–ventral length measured here as dorsal fin to pelvic fin length.

## 4. Conclusions

Craniofacial variation is prodigious across cichlids, with direct impact on feeding strategy and fitness [2,26,27]. Here, we identify the genetic basis for a series of adaptations related to suction feeding versus biting, including overall head proportions, mandible shape, ventral width, and dimensions of the buccal cavity. These phenotypes are not correlated and largely share independent genetic architecture. Our data thus suggest that craniofacial morphologies are likely constrained due to functional demands rather than similar genetics.

## Figures and Tables

**Table 1:** Candidate genes within quantitative trait loci (QTL) intervals. For each interval in Figure 4, we highlight top candidate genes such as transcription factors or components of common developmental signaling pathways. As appropriate, we highlight syndromes that result from mutation of these genes and include craniofacial phenotypes. See Table S4 for a full list of all genes in the interval and text for further explanation of putative roles of some of these genes.

QTL Phenotype	LG	Number of Genes in Interval	Candidate Genes related to Craniofacial Development or Disease
PC2 lateral	2	117	*fgf13, zic3*
Opercle-to-mandible Length	6	573	*fgf8b, fgf20a, p300* (Rubinstein-Taybi syndrome)
Opercle-to-midline width	6	510	*fgf8b, fgf20a, p300* (Rubinstein-Taybi syndrome)
PC2 lateral	6	193	*axin2, smarce1*
Dorsal–pelvic length	7	424	*alx4, hdac10, sema3a, sema3d, sema3e, smad3, smad6, wnt7bb*
PC2 lateral	7	635	*bmpr1bb, foxd1, lhx6, notch1a, pax8, smad4a, smad7, tbx3a*
Preorbital length	7	702	*apc, bmp1, bmp10-like, lhx6, nodal2, fgfr1a, smad2, smad4a, smad7, pax8, tbx3a, tbx5a, tcf4*
PC2 lateral	10	149	*fgf1, fgf13b, spry4, tcf7*
Pelvic–snout length	12	782	*bmp3, fgf10, fgf5, foxd4, lhx6b, ptch1, smarcad1, tbx5*
PC4 lateral	12	246	*dlx5a, dlx6a, hoxa* gene cluster, *smarcc1b, sp8a, twist1a*
Mouth angle	13	300	*dkk1, grem2a, pax2a, sufu, wnt8b*
Eye area	15	158	*bmp2, dll4, med23*
Opercle-to-midline width	15	10	*fzd3a*
Eye area	16	824	*acvr1, acvr1c, bbs5* (Bardet-Biedl ciliopathy)*, dlx1a, dlx2a, epha3, evx2, frzb, fzd5, hoxd* gene cluster, *pou3f3* (Williams–Beuren syndrome)*, satb2, shox, tbx15, tgfbr2l, zic2* (Holoprosencephaly)*, zic5*
Mandible angle	16	504	*frzb, fzd5, pou3f3* (Williams–Beuren syndrome)*, satb2, shox, tbx15, tgfbr2l, zic2* (Holoprosencephaly)*, zic5*
PC3 lateral	17	248	*dkk2*
Preorbital length	17	3	*ephrin type-B receptor 1-B*
Head proportion	20	972	*alx3, hes4, hoxC* gene cluster, *irx7, wnt1, wnt10b, wnt2ba, wnt5a, wnt7a*
Dorsal–pelvic length	20	351	*alx3, hes4, hoxC* gene cluster, *wnt1, wnt10b, wnt2ba, wnt5a*
PC3 lateral	20	226	*hes4*
PC3 lateral	22	246	*col1a2, dlx5, dlx6, hoxA* gene cluster, *smarcc1b*
PC2 lateral	23	556	*crocc2, fgf22, foxd2, lhx8, notch2, prdm5, tgfbr3*
PC4 lateral	23	683	*acvr2a, bmpr2, fgf14, spry2, zeb2* (Mowat-Wilson neurocristopathy)

## Data Availability

Data are accessible at Dryad https://doi.org/10.5061/dryad.4mw6m90cz (accessed on 15 June 2022). These files include phenotypic measures, TPS files for geometric morphometric analysis, and genotypes used for quantitative trait loci mapping.

## Supplementary Materials

The following supporting information can be downloaded at: https://www.mdpi.com/article/10.3390/biology11081165/s1, Figure S1: Shapes indicated by PC scores following geometric morphometric analysis; Figure S2: Genome-wide QTL scans; Figure S3: QTL scans and allelic effects for QTL; Figure S4: Analysis of modularity; Table S1: Statistical significance for phenotypic measures; Table S2: Correlations between all pairs of phenotypic traits in *Labidochromis* × *Labeotropheus* F_2_ hybrids; Table S3: Details of quantitative trait loci (QTL); Table S4: Candidate genes for head shape variation QTL; Table S5: Gene ontology (GO) analysis for enriched processes in candidate genes for QTL.
